# Nanostructured Electrospun Polycaprolactone—Propolis Mats Composed of Different Morphologies for Potential Use in Wound Healing

**DOI:** 10.3390/molecules27165351

**Published:** 2022-08-22

**Authors:** Agnes Chacor de Figueiredo, Javier Mauricio Anaya-Mancipe, Aline Oliveira da Silva de Barros, Ralph Santos-Oliveira, Marcos Lopes Dias, Rossana Mara da Silva Moreira Thiré

**Affiliations:** 1 COPPE/Program of Metallurgical and Materials Engineering—PEMM, Universidade Federal do Rio de Janeiro, Rio de Janeiro 21941-599, RJ, Brazil; 2 Institute of Macromolecules Professora Eloisa Mano—IMA, Universidade Federal do Rio de Janeiro, Rio de Janeiro 21941-598, RJ, Brazil; 3 Brazilian Nuclear Energy Commission, Nuclear Engineering Institute—IEN, Rio de Janeiro 21941-906, RJ, Brazil; 4 Laboratory of Radiopharmacy and Nanoradiopharmaceuticals, Universidade Estadual da Zona Oeste, Rio de Janeiro 23070-200, RJ, Brazil

**Keywords:** electrospinning, morphology structures, propolis, polycaprolactone, drug delivery

## Abstract

This study aimed to investigate different types of morphologies obtained using the electrospinning process to produce a material that enables wound healing while performing a controlled release. Using benign solvents, the authors prepared and characterised electrospun polycaprolactone mats loaded with propolis, a popular extract in traditional medicine with potential for skin repair. Different morphologies were obtained from distinct storage periods of the solution before electrospinning to investigate the effect of PCL hydrolysis (average diameters of fibres and beads: 159.2–280.5 nm and 1.9–5.6 μm, respectively). Phytochemical and FTIR analyses of the extract confirmed propolis composition. GPC and viscosity analyses showed a decrease in polymer molecular weight over the storage period (about a 70% reduction over 14 days) and confirmed that it was responsible for the nanostructure diversity. Moreover, propolis acted as a lubricant agent, affecting the spun solutions’ viscosity and the thermal properties and hydrophilicity of the mats. All samples were within the value range of the water vapour transpiration rate of the commercial products (1263.08 to 2179.84 g/m^2^·day). Even though the presence of beads did not affect the propolis release pattern, an in vitro wound-healing assay showed that propolis-loaded mats composed of beaded fibres increased the cell migration process. Thus, these films could present the potential for use in wound dressing applications.

## 1. Introduction

One of the largest organs in the human body, the skin is essential to homeostasis. In addition to protecting against physical and chemical injuries, it also acts as a barrier against pathogens, controls water loss, is a sensory receptor, protects against ultraviolet radiation, converts precursor molecules into active vitamin D, contributes to body thermoregulation, and excretes substances [1]. For that reason, it is essential to recover the original tissue and its function when it is injured, avoiding function loss in the scarring process [2].

Therefore, advances in regenerative medicine and the need for new alternative methods for wound healing promote the advent of new materials capable of mimicking skin extracellular matrix structures and their properties [3]. These new biomaterials can be used in several critical skin wound regeneration applications, such as in burns, injuries caused by trauma accidents, and even chronic wounds.

Among several methods for scaffold production, electrospinning is a technique capable of creating nanostructured mats using polymer solutions. The system consists of an infusion pump, a high voltage source, a metallic collector, and a syringe with polymer solubilised in a conductive solvent. These nanostructured mats are effortlessly produced and are able to replicate skin extracellular matrix structures [4,5,6]. Aside from acting as a barrier to pathogens while the tissue is repairing itself, they also provide a large surface area to volume ratio, which can be favourable for local drug delivery, and have ideal porosity for gas transition [7]. However, many variables must be evaluated for the desired morphology acquisition, including the molecular weight, polymer concentration, viscosity, surface tension, and conductivity of the polymer solution used. Furthermore, process variables, such as voltage and flow rate, and environmental factors, such as temperature and humidity, should be considered as well [8,9]. In the literature, there is a focus on obtaining uniform fibres. However, some articles report that the presence of bead-like structures—considered as artefacts or by-products of fibres—make electrospun mats more effective in drug loading and sustained release [10,11,12]. In this context, nanoparticles have also been widely used as a vehicle for the delivery of drugs and biomolecules [13].

Among popular polymers used in the electrospinning method, there is polycaprolactone (PCL) due to its easy spinnability. PCL is a Food and Drug Administration (FDA)-approved, semi-crystalline polyester with potential for biomedical applications and delivery systems because of its biocompatible and biodegradable characteristics. It is one of the most used biopolymers for skin regeneration and is easy to process in various formats, producing films, mats, membranes, or fibres [14,15].

Propolis is a resinous bee mixture widely used in traditional medicine. Its composition varies according to the honeybees’ food source and may contain more than 300 components [16]. Cheap and easy to find, its extract has antioxidant, anti-inflammatory, anti-microbial, and healing properties due to the presence of phenolic compounds and flavonoids in its composition [7,17,18,19,20]. In an ideal concentration, propolis has the potential for skin repair aid and contributes to restraint pathogens. Oliveira et al. [21], for example, developed a hydrophilic PVA/CMC dressing with propolis for burn treatment. More recently, Stojko et al. [22] and Alberti et al. [23] produced PLA and PVA electrospun fibres both loaded with propolis, and Salimbeigi et al. [24] produced nanofibres of PCL/propolis using chloroform/methanol (7:3) as system solvent also for wound-healing applications.

PCL electrospinning has been commonly carried out using halogenated solvents such as 1,1,1,3,3,3-Hexafluoropropan-2-ol (HFIP), chloroform, and dichloromethane (DCM), as they have a good affinity for PCL, in addition to giving the solution a good electrical permittivity, helping with spinning [25,26,27]. Such solvents, however, present high toxicity to the organism’s cells, posing health risks. For this reason, the use of greener solvent systems such as acetic acid and formic acid for PCL solubilisation has been reported [28,29,30,31,32,33,34,35,36]. This mixture of solvents in several proportions was evaluated, and its efficacy in PCL electrospun fibres’ production was proved. Moreover, it was reported that the acetic acid/formic acid system enables cost and toxicity reductions compared to more conventional solvents. While formic acid contributes to the solution’s electrical permittivity, acetic acid is mainly responsible for polymer solubilisation [30]. This study aims to contribute to the advances in skin tissue engineering by producing and evaluating PCL+ Propolis (Prop) electrospun mats for potential use in wound healing. Different morphologies (fibres, beaded fibres, and beads) of PCL + Prop mats were studied parallel to pure PCL samples. The physicochemical properties of the solutions and the electrospun mats were analysed. In addition, the potential of the mats to accelerate wound healing was evaluated using an in vitro scratch wound-healing model.

## 2. Results and Discussion

### 2.1. Propolis Extract Chemical Composition Analysis 

As the composition of propolis may vary depending on honeybees’ food source, it is relevant to analyse its phytochemical composition, as there may be a presence or absence of some compounds. 

First, a simple qualitative analysis verified the presence of typical components of the propolis extract (images available in Supplementary Materials, Figure S1). Based on the protocols of [37], the existence of flavonoids and phenolic compounds was confirmed by the change of colours after the addition of 5% wt. AlCl_3_ and 2% wt. FeCl_3_ solution on the propolis extract used in this study (yellow for flavonoids and dark blue for phenolic compounds, respectively).

**Figure 1:**
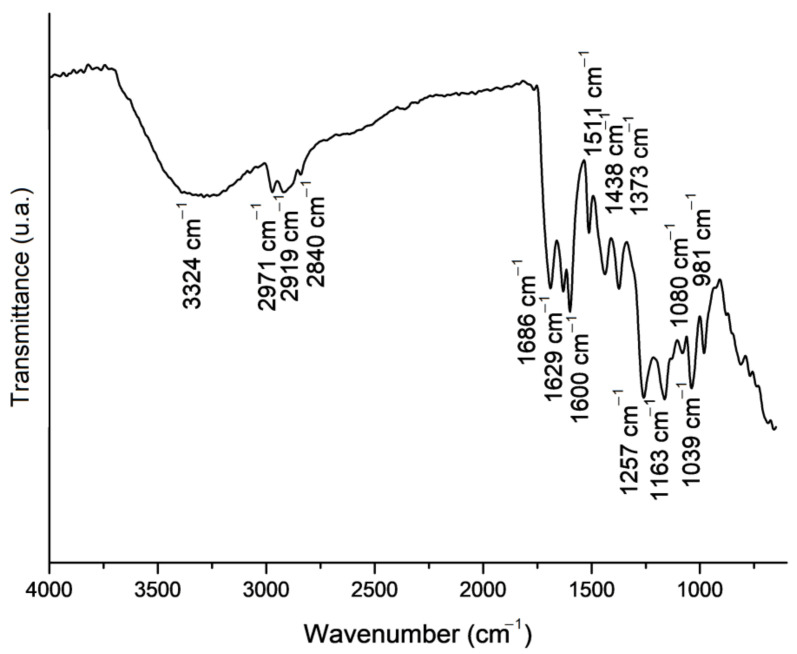
Fourier-Transform Infrared Spectroscopy bands related to the propolis extract.

For a more in-depth study of the composition, an FTIR analysis was performed, and Figure 1 shows the spectrum related to the extract. The broad band at 3324 cm^−1^ is assigned to O–H group stretching vibrations, as well as to C–H, H–C of aromatics, O–H of flavonoid compounds, and N–H of amino acids [38]. Bands 2971 and 2919 cm^−1^ could be related to the asymmetric stretch of CH_2_ in ethanol, while 2840 cm^−1^ is assigned to its symmetric stretching. The 1689, 1629, and 1600 cm^−1^ bands could be related to the stretching vibrations of C=C and C=O groups in flavonoids and N-H asymmetric stretch vibrations of amino acids [39]. The band at 1511 cm^−1^ could be attributed to flavonoids and aromatic rings (deformations and stretching of C=C aromatic groups) [40]. 1373 cm^−1^ is associated with scissoring vibrations of C-H groups in hydrocarbons and flavonoids [41]. The 1257 cm^−1^ band could be assigned to C-O groups in polyols, such as hydroxy-flavonoids, or to wagging vibrations of C-H groups in phenolic compounds [38]. The band at 1163 cm^−1^ is related to C-O stretching vibrations in lipids and C-OH bending vibrations in tertiary alcohol groups [39,40]. The 1080 cm^−1^ band could be attributed to the O-H of stilbenes, steroids, fatty acids, carboxylic acids, and secondary alcohols. This band is also assigned to C-O groups in flavonoids and terpenes [38]. The band at 1039 cm^−1^ represents the stretching vibrations of C-O ester groups and also primary and secondary alcohols [40]. The 981 cm^−1^ band is associated with the scissoring vibrations of CH_3_ in esters [41].

The FTIR analysis confirmed the presence of some active compounds which are relevant to wound-healing process improvement. Flavonoids, for example, have anti-inflammatory properties and assist in tissue regeneration, as they contribute to re-epithelialisation and angiogenesis matrix remodelling. They also have antioxidant, anti-cancer, and anti-viral activities [40,42]. Likewise, terpenes also have anti-microbial activity that could facilitate wound healing [43], and phenolic compounds also have anti-inflammatory and antioxidant activity, in addition to stimulating collagen production in fibroblasts [44].

### 2.2. Effect of Storage Time on PCL Molecular Weight and Spun Solution Viscosity

PCL and PCL + Prop solutions in acetic acid and formic acid (9:1) were prepared. Prior to the electrospinning process, these solutions were stored at 35 °C for up to 28 days to evaluate the polymer exposure to the solvent mixture.

**Figure 2:**
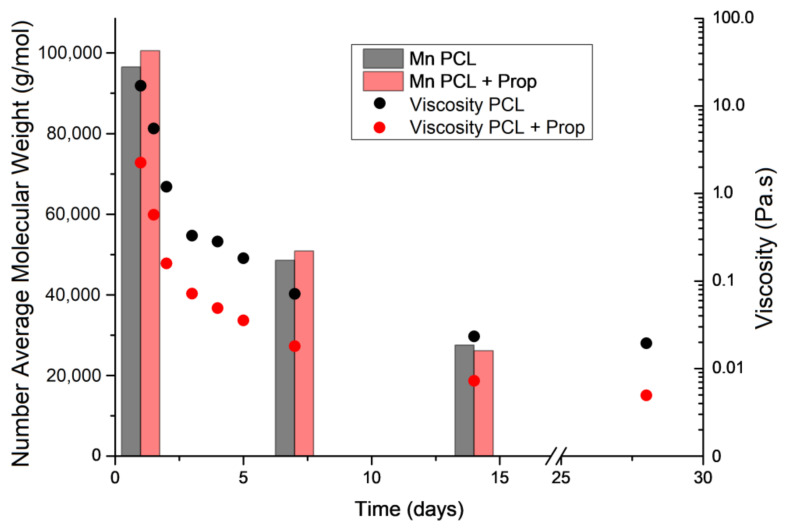
Number average molecular weight and viscosity for PCL and PCL + Prop solutions after days of dissolution.

Figure 2 shows viscosity, as well as polymer molecular weight, which are two of the most important parameters in the electrospinning process as they describe the entanglement capacity of polymer chains that will result in fibre formation. Viscosity analysis was carried out on the solutions before processing, while number average molecular weight (Mn) analysis was performed on electrospun mats (using solutions with 0, 7, and 14 days of storage time). 

The viscosity values of both solutions decreased over time, as exponential-like behaviour was attributed to PCL hydrolysis due to exposure to the AA/FA solvent system. At all times tested, up to 28 days, the PCL + Prop solution was always less viscous than the pure PCL one, which agrees with the behaviour observed for propolis/PVP solution viscosity evaluated by Moghaddam et al. [45]. On the other hand, gel permeation chromatography analysis of the electrospun mats on days 0, 7, and 14 indicated that Mn values for PCL in PCL solution and in PCL + Prop solution had an approximately 50% decrease with each week spent in storage. These results corroborate the viscosity values, although they imply that the propolis extract addition does not affect the polymer Mn but acts as a lubricating agent, which increases the free volume and the mobility of polymer chains [46].

According to Dias et al. [47], the viscosity of the solution is related to the entanglement of polymer chains. The reduction in these entanglements, whether due to low polymer concentration or low molecular weight, affects the spinning process as a result of the Taylor cone’s destabilisation, which can generate defective fibres or even only bead mats [8,28]. Nevertheless, Lavielle et al. [31] and Gil-Castell et al. [33], although evaluating PCL in different concentrations and using an AA/FA solvent system in a different ratio, reported similar decreases in molecular weight within eleven and five days, respectively. It was suggested that when using the mixture as a solvent, PCL is subjected to degradation via acid hydrolysis [31], which generates instability in electrospinning.

### 2.3. Morphology and Fibre Diameter in the Electrospun Mats

**Figure 3:**
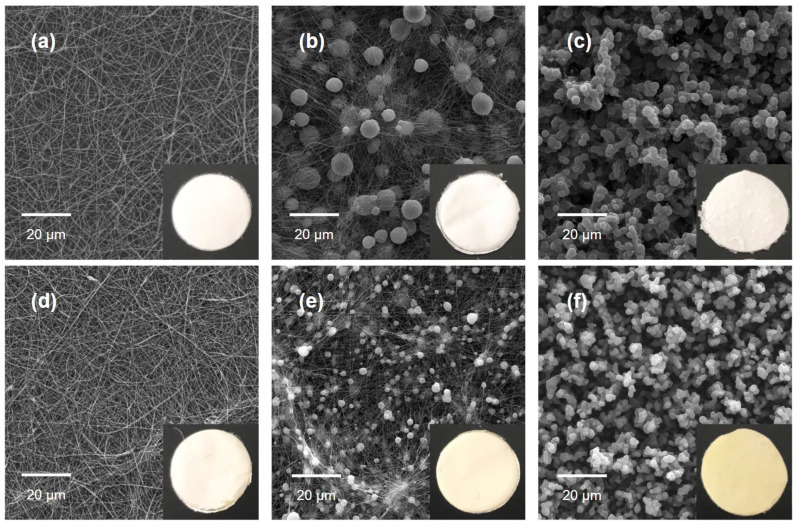
Scanning electron microscopy images and macrographs (disks of 1.5 cm diameter) of electrospun mats from solution storage for different periods: (**a**,**d**) 0 day (PCL fibres, PCL + Prop fibres); (**b**,**e**) 7 days (PCL beaded fibres, PCL + Prop beaded fibres); (**c**,**f**) 14 days (PCL beads, PCL + Prop beads).

Solutions of PCL and PCL + Prop after different storage periods were electrospun by varying the flow rate and applied voltage (data not shown). Table 1 presents the best conditions for morphology acquisition, generating a total of six samples of PCL and PCL + Prop mats. The morphology of the mats was evaluated using scanning electron microscopy (SEM) (Figure 3), and the diameter of the structures was quantified using an image treatment carried out with SizeMeter 1.1 software (Table 2). SEM images of the mats with higher magnifications and histograms of diameter measurements are available in the Supplementary Materials (Figures S2–S4).

As observed in Figure 3, PCL and PCL + Prop solutions were immediately electrospun after solubilisation, generating uniform and continuous fibres, while 7-day storage resulted in a decrease in the average fibre diameter and the occurrence of beads. On the other hand, the increase in the storage time to 14 days only generated bead mats, which was a morphology also previously reported in [48,49]. No surface changes were observed in the structures of all six samples.

The differences among morphologies could be explained by the solution’s exposure time to the solvent system as it changed the polymer molecular weight and viscosity. As reported by Haider et al. [4], a decrease in molecular weight, and consequently in viscosity, made the Taylor cone less stable as the polymer chains are less entangled and susceptible to breaking due to the voltage and surface tension before reaching the collector, which will entail gross defects in electrospun mat morphology (the appearance of beads and a higher standard deviation of fibre diameters). At 14 days of exposure, due to the significant decrease in PCL molecular weight, the electrospinning process is differentiated and usually referred to as electrospray [50]. 

Once defects in fibres are present, there is also a drastic decrease in fibre diameter (compared to only fibre samples), as seen in Figure 3b,c. This is related to the use of low-concentration and/or low-molecular-weight polymer solutions. In both cases, it relates the density of chain entanglements to the stability of the Taylor cone [30,51].

Abel et al. [52] showed PCL hydrolysis in an aqueous solvent system of acetic acid and formic acid and demonstrated that the present water acted as a catalyst for said hydrolysis compared to the PCL solution in an acetic acid and 98% formic acid mixture. However, in our study, the significant hydrolysis of PCL was registered, which drastically decreased the molecular weight of the polymer and increased the polydispersity, even though there was no water in the acetic acid/formic acid solvent system (pH < 3) used. Even so, this hydrolysis is in conformity with the data reported in the literature for the acid degradation of this type of polyester [53].

Moreover, the electrospun mats’ macrographs also allow an increase in colour in PCL + Prop samples to be observed as the solution storage time increases, especially in bead mats. The yellowness is characteristic of the natural extract, which suggests that small PCL chains tend to interact less with the propolis.

From these analyses onward, electrospun mats prepared from stored solutions (0, 7, and 14 days) are designated as “PCL fibres”/“PCL + Prop fibres”, “PCL beaded fibres”/”PCL + Prop beaded fibres”, and “PCL beads”/”PCL + Prop beads” mats, respectively.

### 2.4. Thermal Behaviour of Electrospun Mats

**Figure 4:**
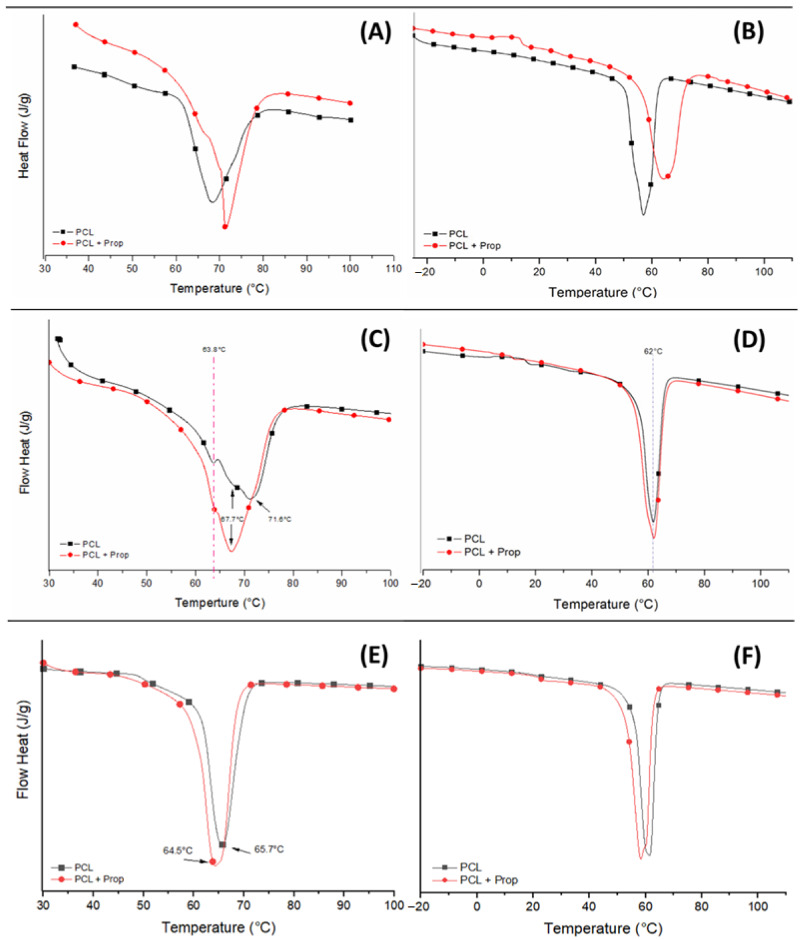
DSC curves of electrospun PCL and PCL + Prop mats: first and second heating, respectively. (**A**,**B**) Fibres, (**C**,**D**) beaded fibres, and (**E**,**F**) beads.

The thermal behaviour of the PCL and PCL + Prop electrospun samples obtained from solutions stored at 0, 7 and 14 days were evaluated using differential scanning calorimetry (DSC) (Figure 4). The melting temperatures (T_m_), enthalpies (ΔH_m_), and degree of crystallinity (X_c_) for each thermal transition in the first and second heat cycles are displayed in Table 3. 

In Figure 4A, fibres with propolis incorporation presented two endothermic peaks. The one allocated at 71 °C was attributed to the melting of a population of more perfect crystals compared to the crystal population on pure PCL fibres melted at the same point. On the other hand, the second peak close to 64 °C was attributed to another population of crystals not seen on the pure PCL fibre thermogram. Therefore, this peak could be assigned to PCL and propolis interactions, which favoured the formation of this more imperfect type of crystals [54].

For the electrospun samples exposed to 7 days of storage (beaded fibre morphology), the presence of two endothermic peaks was also observed, showing the presence of two populations of crystals as well (Figure 4C). However, the population melting at approximately 64 °C showed a higher intensity compared to the crystals of PCL + Prop fibres, demonstrating a formation of more crystals that may have been due to the packaging of smaller chains due to hydrolysis of the PCL in acetic acid. 

Finally, the thermal transitions of the electrospun beaded structures (Figure 4E) demonstrated a greater bundling of the chains, attributed to a lower molecular weight, as seen in the GPC analysis (Figure 2), which only generates a population of crystals for both PCL and PCL + Prop at approximately 65 °C. Comparing the first and the second heatings of both types of beads and taking into account their X_c_, there seems to have been no interactions between the lower-molecular-weight PCL and the propolis [55,56], which could also explain why the PCL + Prop beaded mat presented such an intense yellow colour and corroborates the interpretation of the viscosity analysis. 

**Figure 5:**
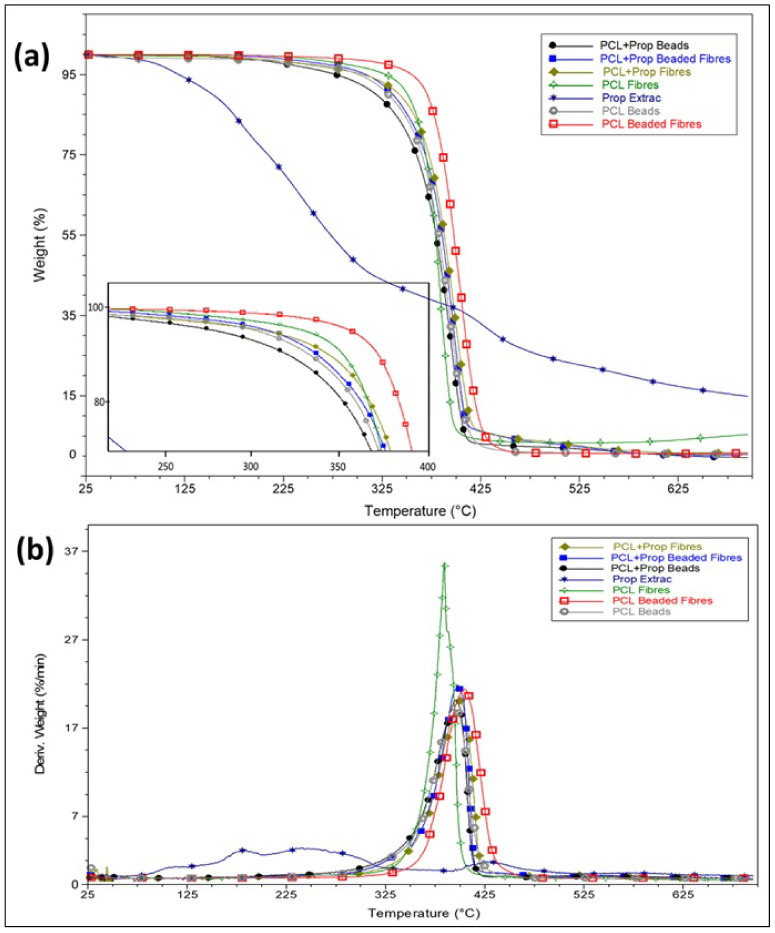
Thermal behaviour: (**a**) TG curves and (**b**) DTG of PCL and PCL + Prop electrospun mats.

Thermogravimetric analysis was performed to analyse the thermal stability of the electrospun mats (Figure 5). The electrospun films only had one degradation event among 383–404 °C, a range typically attributed to PCL degradation [28].

There were no thermal events before PCL degradation, indicating that the mats did not present residual solvents, which is promising for materials intended to be applied as wound dressings. Contrasting to Abel et al. [52], who also worked with PCL solutions in AA/FA in mixtures and electrospinning (as mentioned in Section 2.3), the mat with the lowest stability was the PCL fibre one and not the sample with lower molecular weight (beads mat). Furthermore, the one with the highest temperature degradation was the beaded fibre PCL mat (morphology registered with lower stability in their study). The difference between the results showed in our study and those reported by Abel et al. [52] could be related to the absence of water in our solvent system, as already explained before. 

Although the PCL fibre sample had a higher molecular weight, the electrospun mat also had a lower thickness (see Section 2.6) and, combined with its higher area/volume ratio morphology, may have been shifted to lower degradation temperatures. The PCL beaded fibre film had higher stability, possibly due to its bead morphology (mean diameter of 5.6 um). The formation of more crystals, observed from the DSC analyses, would require more energy for thermal degradation. Moreover, the beads’ size in this sample was large enough to have a degradation temperature similar to the PCL pellets, as seen in [28].

### 2.5. Wettability Analysis 

**Figure 6:**
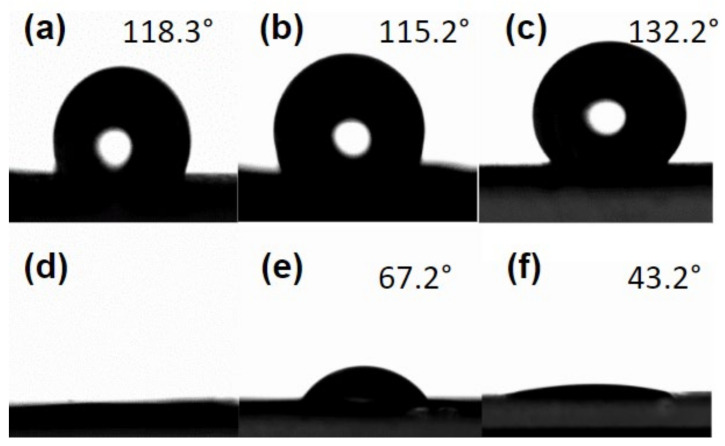
Water contact angle for electrospun samples with morphological variations. (**a**) PCL fibres; (**b**) PCL beaded fibres; (**c**) PCL beads; (**d**) PCL + Prop fibre; (**e**) PCL + Prop beaded fibres; (**f**) PCL + Prop beads.

The wettability of the samples was evaluated through water contact angle (WCA) measures, as shown in Figure 6. In this analysis, one water drop was allocated on every film with and without propolis extract with morphology variations.

Even though PCL electrospun mats presented slight variations in the contact angle attributed to the surface roughness related to the specific morphology of each mat, they still confirmed PCL’s hydrophobic nature [57]. Although PCL has good biocompatibility, this poor wettability due to low surface energy can hinder cell adhesion [58]. Thus, when designing PCL-based dressings, this should be a major consideration, since PCL is a hydrophobic polymer [15].

On the other hand, the addition of propolis extract to the mats resulted in a significant decrease in contact angle measurements, which indicated an increase in the wettability of the mats. The fibre mats WCA could not even be measured as the water drop deposited was immediately absorbed. This more hydrophilic nature can be attributed to the composition of propolis extract that, although varying according to its origin, has a high prevalence of terpenoids, phenolic acids, and flavonoids [59,60]. These compounds have a polar character due to their oxygenated groups, which favours interaction with water and reduces the average contact angle on the samples. These results are in accordance with the studies of Li et al. [61], where the authors evaluated different coatings of polydopamine (rich in polar groups) in PCL electrospun mats, generating changes in PCL samples, from hydrophobic to super hydrophilic.

The WCA for the PCL + Prop beaded fibres mat was larger than the measurement for the PCL + Prop beads mats, which can be explained by the diameter of the structures found in both samples. In addition to fibres, the beaded fibre mat had beads approximately twice the size of the structures in the bead mat. This result is in agreement with Jia et al. [62]: larger structures form more prominent cavities and trap more air between the water drop and the mat surface, which results in a more hydrophobic state.

### 2.6. Water Vapour Transpiration Rate (WVTR) Analysis

**Figure 7:**
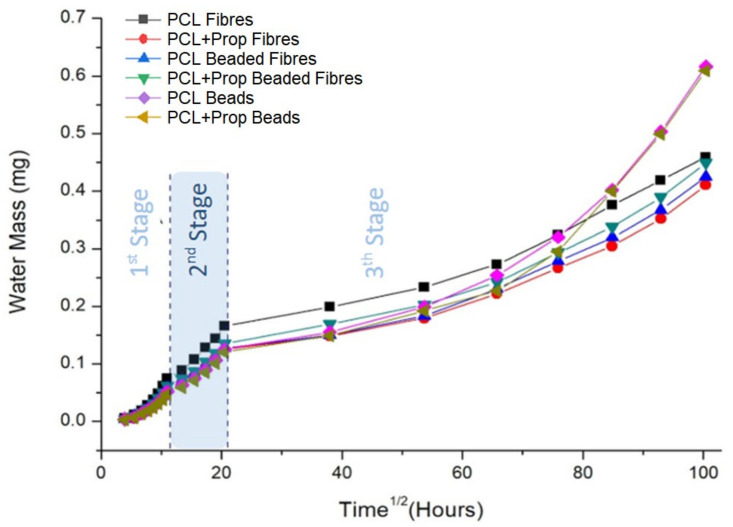
Water vapour flow (J) in electrospun mats with morphology variations.

Water vapour transpiration rate (WVTR) analysis evaluates the moisture conditions that a potential dressing can provide for a wound. In this study, WVTR analyses were carried out in electrospun samples with and without propolis, evaluating the water vapour across the mats, as represented in Figure 7. From these values, we calculated the WVTR, shown in Table 4.

In this study, we used the second stage as a reference to the diffusion process in the mats, described by Fick’s law, and calculated the mass variation over time [63]. Later calculations were also made to find their transpiration flow. Thickness was used to normalise the values of WVTR. 

PCL fibres had a lower thickness than other mats (Table 4). The zero-day-storage PCL solution had the highest viscosity, which could have made it difficult for the infusion pump to push the syringe plunger. Therefore, less material could have reached the collector in 2 h, generating a thinner mat. Since the other spun solutions had lower viscosity, we suggest that their thickness could be related to the organisation and packaging of fibres and beads. In these cases, mixed structures (beaded fibres) could be more difficult to pack than single structures (PCL Beads, PCL + Prop fibres, or PCL + Prop Beads), which could lead to thicker mats.

The rates of electrospun mats ranged from 1263.08 to 2179.84 g.m^−2^ per day, which are values within the range of WVTR of commercial dressings characterised mainly by large porosity, such as foams [64]. Thus, all samples achieved permeability values within the effective wound treatment range reported in the literature, as the rates on healthy skin are 204 g/m^2^ per day and on wounded skin, 279–5138 g.m^−2^ per day [65], and possibly could prevent exudate accumulation as well as excessive dehydration.

### 2.7. Propolis Release Analysis

**Figure 8:**
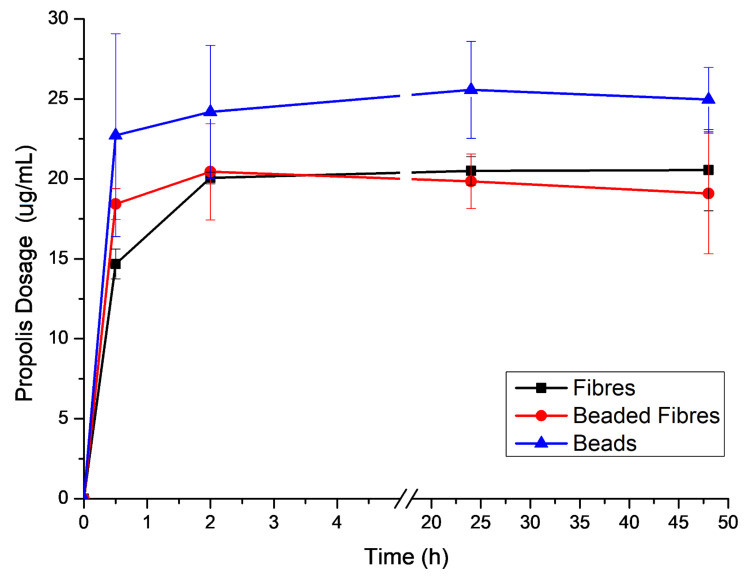
Propolis release profile in the function of the delivery time for the different morphologies.

To evaluate the delivery of the propolis encapsulated in the electrospun PCL structures, a release assay was performed for different times up to 48 h, as shown in Figure 8. This was quantified using a UV-Vis spectrometer. PCL + Prop samples were evaluated for 48 h in saline solution (0.9% wt. NaCl) at 37 °C and 100 rpm in a Shaker incubator. The calibration curve is available in the Supplementary Materials (Figure S5).

Electrospun mats have good properties for potential biological applications, such as a large surface area to volume ratio and high porosity that facilitates permeation. Those characteristics, however, also facilitate diffusion. Therefore, a similar burst release tendency was observed in all mats in the first 30 min of the trial, even though several works reported that the presence of beads in the electrospun structures could result in more sustained delivery time due to the increase in the diameter of the mat and/or layers in which said active components would have to diffuse through for release [10,11,12]. However, it seems that PCL + Prop fibres only reached their equilibrium concentration release after 2 h, which was equal to those of beaded fibres. Electrospun mats composed only by beads presented a higher maximum concentration of propolis released. 

Furthermore, at 37 °C, PCL is above the glass transition temperature, which increases polymer chain mobility [66]. As propolis is more hydrophilic and has more affinity with the aqueous medium than with the polymer matrix and acts as a lubricating agent, enlarging the PCL free volume, extract diffusion is favoured. These features promote the burst effect in vitro, as also shown by da Silva et al. [67] and Maver et al. [68]. 

Besides that, the PCL + Prop beads morphology sample showed a slight increase in the release of propolis, which could also have been related to the porosity of the mat. These mats also immobilised a slightly higher amount of propolis than those containing fibres (fibres and beaded fibres). Although there is no evidence of good interaction between extract and polymer as seen previously, the larger pores of the beaded mats could have allowed better penetration and contact with saline through the structure, which may have favoured the diffusion process.

**Figure 9:**
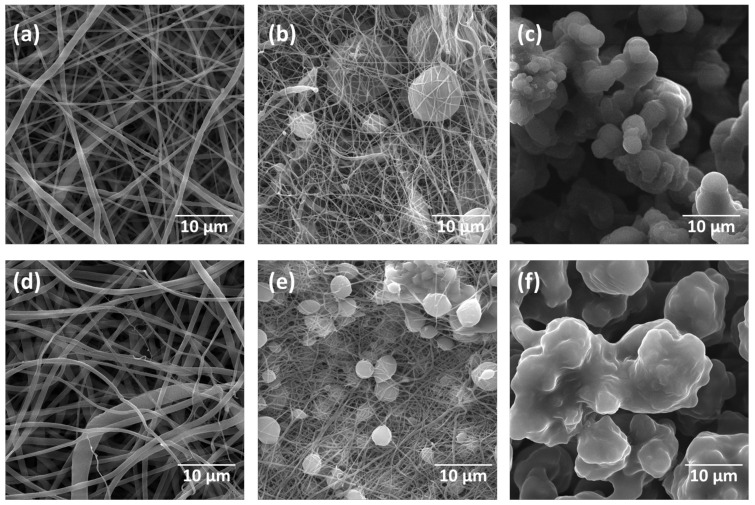
Scanning electron microscopy images of membranes with different morphologies after swelling and propolis release (48 h). PCL (**a**–**c**) and PCL + Prop (**d**–**f**).

Furthermore, some works described that drug delivery from nanostructured mats could also be influenced by other mechanisms besides diffusion, such as swelling [69]. Likewise, this was probably present in the PCL mats’ release pattern, as analysed using SEM after the release test (Figure 9). Samples’ diameters were compared before and after the release test. These data are presented in Table 5. 

Both PCL and PCL + Prop fibre samples showed an increase in the diameter of their structures after immersion in saline solution for release testing, indicating a swelling pattern (see histograms in Figure S6, Supplementary Materials). Similar results were obtained in the studies of Reshmi et al. [70], in which PCL and PCL + nano chitosan fibres become swollen after 28 days in saline solution.

On the other hand, the fibres’ diameter in beaded fibre mats also increased, but on a smaller scale compared to only fibre mats. Their beads, however, did not seem to undergo a significant increase in diameter, which leads to the understanding that diffusion may have contributed to the release of propolis in this mat structure more than the swelling mechanism.

Additionally, the bead-only samples demonstrated a visible swelling pattern only on the pure PCL mats. In the case of PCL + Prop samples, the structures were not measurable, as they appear to have melted, which suggests that the release of the extract—as it acts as a lubricating agent in a lower-molecular-weight sample—may have abruptly altered its morphology.

### 2.8. In vitro Wound-Healing Assay

**Figure 10:**
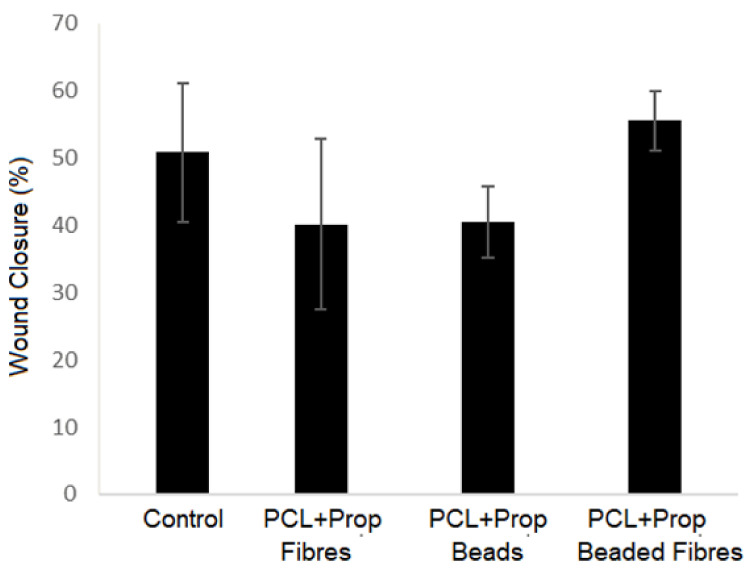
Quantitative analysis of fibroblasts migration area after 24 h of wound healing in the presence of PCL/Prop mats. Cells cultivated without any film were used as control.

**Figure 11:**
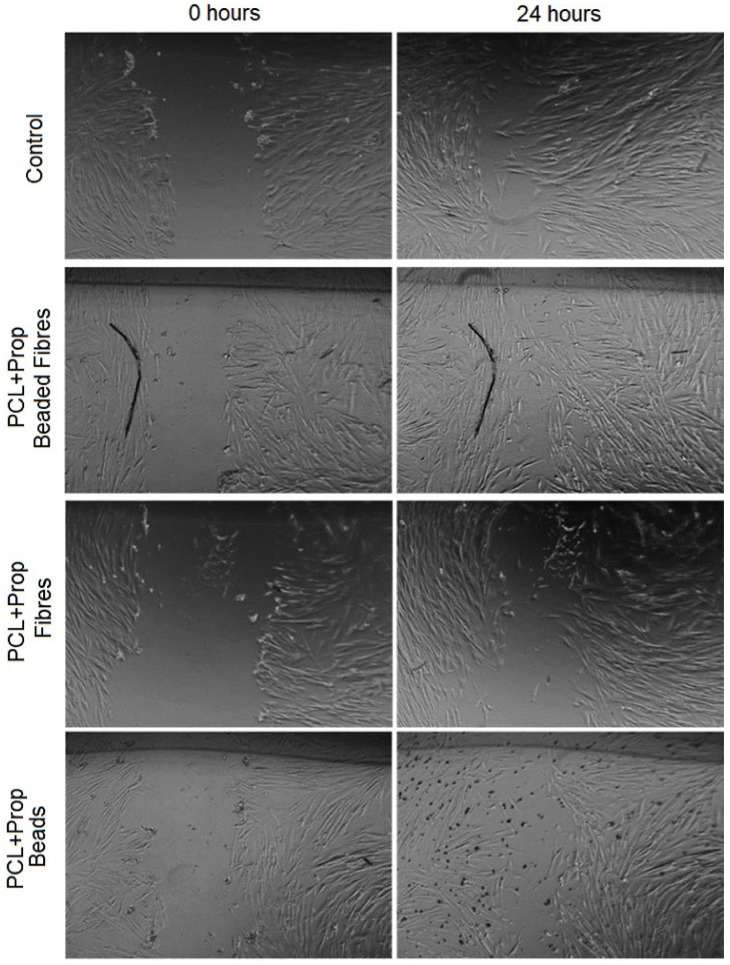
Evaluation of the scratch wound-healing assay using human gingival fibroblast (FGH) cell staining with microscopic inspection of cells stained immediately after scratching (0 h) and after 24 h of wound healing.

The scratch wound assay was used to investigate cell migration in the presence of the samples after a gap in a confluent monolayer of cells was created to mimic a wound. Since cell migration is a crucial step for wound healing, this test could be used to estimate the in vitro capacity of the films to accelerate healing. The results are shown in Figure 10 and Figure 11.

PCL + Prop beaded fibre film had the most prominent results, since it could increase the healing in 24 h by fastening the migration cell to the gap (Figure 10 and Figure 11). On the other hand, the presence of propolis in fibres mats and in beaded mats showed no significant effect on the wound-healing rate as compared to the control. 

The wound-healing effect of propolis has been demonstrated in several in vitro and in vivo studies using different nanosystems for propolis delivery [23,71,72,73,74,75,76,77]. One of the main mechanisms in wound healing promoted by propolis relies on the anti-microbial properties, (i) reducing biofilm generation and (ii) accelerating the healing processes [74,78]. Additionally, propolis has antioxidant properties due to the presence of flavonoids, phenolic acids, or terpenoids [79,80] and has an anti-inflammatory effect by inhibiting the nitric oxide in the macrophages [81]. 

For example, Alberti et al. [23] observed a wound site reduction of 68% after 7 days of in vivo and in vitro treatment using nanofibres of polyvinyl alcohol (PVA) and propolis. However, to our knowledge, no study has previously been conducted with PCL + Prop electrospun mats. Furthermore, Karizmeh et al. [57] demonstrated that electrospun PCL/chitosan fibres embedded with propolis potentiate the wound-healing effect. Finally, Lesmana et al. [82] stated that propolis improves the wound-healing properties of biomaterials without negative effects on the physicochemical properties of composite biomaterials. 

Accordingly, our data demonstrated that the presence of propolis in a mixed nanostructured form increased the migration process. Since the propolis release pattern of the mats was very similar, considering the propolis extract used, the positive response to FGH cells could be more related to the morphology, in particular, surface roughness and wettability, than to the presence of propolis. However, further studies should be conducted to evaluate the effect of the propolis concentration on the cell response to the PCL + Prop beaded fibre sample.

## 3. Materials and Methods

### 3.1. Materials

Film samples were produced using PCL pellets by Sigma-Aldrich Brazil (Mn: 80,000 g/mol, degree of hydrolysis of 98 mol%) purchased from Sigma-Aldrich, São Paulo, SP, Brazil, while glacial acetic acid (AA) and 98% pure formic acid (FA) were purchased from Vetec Química Fina LTDA, Rio de Janeiro, RJ, Brazil. Alcoholic extract of Brazilian green propolis (minimum of dry extract of 11% *w*/*v*) was purchased from Apis Flora Indústria Brasileira, São Paulo, SP, Brazil.

### 3.2. Methods

#### 3.2.1. Propolis Extract Characterisation

Phytochemical qualitative analysis was performed based on the protocols of [37] for confirmation of the presence of flavonoids and phenolic compounds. For phenolic compounds, the extract sample was dripped on filter paper, followed by drops of 2% wt. FeCl_3_ solution. The appearance of a dark blue spot would indicate the presence of phenolic compounds. On the other hand, for flavonoids, the extract sample was dripped on filter paper, followed by the addition of a 5% wt. AlCl_3_ solution. The appearance of a yellow colour under UV light (365 nm) would indicate the presence of flavonoids.

The chemical composition of the propolis extract was further evaluated using Fourier-Transform Infrared Spectroscopy (Perkin Elmer, mod. Spectrum 100, Boston, MA, USA) analysis equipped with an attenuated total reflectance (ATR) accessory. Prior to the analysis, the extract was dried in an oven at 35 °C for 12 h, which generated a sample in film format. The analysis was performed in the region of 4000–650 cm^−1^, with 64 scans and a resolution of 4 cm^−1^.

#### 3.2.2. Polymeric Solutions 

Two 30% *m*/*v* of PCL solutions were prepared using acetic acid and formic acid (9:1) as solvent systems according to Mancipe et al. [28]. Both were solubilised at room temperature for 12 h with constant mechanical stirring. 15% *v/v* of propolis extract was added to the second solution [83]. Subsequently, the solutions were immediately subjected to electrospinning or stored at 35 °C for up to 28 days to evaluate the polymer exposure to the solvent mixture.

The spinning conditions (Table 6) focused mainly on the flow rate, voltage, and solution storage time to obtain optimal values for the production of PCL mats and PCL + Prop mats with three different morphologies (fibres, beaded fibres, and beads). Then, once the electrospinning conditions were established, the solutions were electrospun for 2 h to obtain mats with adequate thickness for easy manipulation and subsequent characterisation. 

#### 3.2.3. Viscosimetry and Gel Permeation Chromatography (GPC) Analyses 

Viscosity analysis of the polymeric solutions was performed with a rotational test on the Physica MCR 501 Anton Paar rheometer (Graz, Austria) with cone plate geometry; gap: 0.1 mm; 0–150 (1/s) and measured at 1 (1/s). The solution viscosity was subjected to daily analysis for up to 7 days and then at 14 and 28 days of storage time. The average number molecular weight was evaluated using electrospun films obtained from solutions stored for 1, 7, and 14 days with Shimadzu LCSolution gel permeation chromatography (GPC) equipment (Kyoto, Japan) to corroborate the viscosity analysis. Approximately 4 mg of electrospun samples was analysed using chloroform as the solvent and an injection volume of 2 µL. For the molecular weight determination, a calibration curve based on monodisperse polystyrene was used.

#### 3.2.4. Scanning Electron Microscopy (SEM)

The electrospun mats were golden coated in sputter equipment (Denton Vacuum—Desk V, Moorestown, NJ, USA) for 120 s at 30 mA and a vacuum of 50 mTorr and evaluated using VEGA3 TESCAN (Brno-Kohoutovive, Brno, Czech Republic) scanning electron microscopy with 15 kV acceleration for morphology evaluation. Fibre and bead diameters were obtained using Size Meter 1.1 software (80 measurements for each mat). Since different lots of each type of mat were produced, their morphology was confirmed using SEM before any other analysis.

#### 3.2.5. Thermal Characterisation

Differential scanning calorimetry (DSC) was carried out in a Hitachi—DSC 7020 Thermal Analysis system to study the thermal behaviour of the electrospun mats. First, 7.0 mg of each sample was subjected to two heating cycles and one cooling cycle, which were carried out at a rate of 10 °C/min using nitrogen atmosphere with a flow rate of 50 mL/min. The first heating cycle was conducted from 25 to 90 °C, followed by a cooling cycle to 0 °C and subsequent heating from 0 to 90 °C. The degree of crystallinity of the material (X_c_) was calculated by Equation (1).

(1) $$X_{c}=\frac{\Delta H_{f}^{}}{\Delta H_{f}^{o}}$$

where Δ*H_f_* corresponds to the melting enthalpy of the endothermic peak of the DSC thermogram (second heating), while Δ*H^o^_f_* = 151.7 J/g is the theoretical melting enthalpy for a 100% crystalline PCL sample [32].

Furthermore, the thermal stability and weight loss of the propolis extract and PCL and PCL + Prop electrospun mats were evaluated via thermogravimetry analysis using Shimadzu TGA-50 equipment with a heating range of 25 °C to 700 °C and a heating rate of 10 °C/min under N_2_ atmosphere. Prior to the analysis, the extract was dried in an oven at 35 °C for 12 h, which generated a sample in film format.

#### 3.2.6. Wettability Analysis 

The contact angle was measured with the Ramé-Hart NRL A 100-00 goniometer (Cedar Knolls, NJ, USA) to evaluate the wettability of the electrospun mats. Distilled water (2 µL) was deposited on the surface of a 1 cm × 4 cm sample at room temperature. The analysis was made in triplicate (*n* = 3), and the reported contact angle value corresponded to the average of 100 measurements performed on each sample (1 measurement/second).

#### 3.2.7. Water Vapour Transpiration Rate Analysis

Furthermore, a water vapour permeation test was performed on electrospun samples for seven days following ASTM D 1653. A permeability cup (Payne cup) filled with 10 g of distilled water and the electrospun film sample fixed on its opening was placed inside a closed glass chamber at room temperature, along with a sodium pentoxide (C_5_H_11_NaO) reservoir. Evaporation through the films was monitored by measuring the weight loss of the cup at initial intervals of 15 min until the second hour, then at intervals of 1 h until the seventh hour, and then, finally, at intervals of 24 h until the end of 7 days. Weight loss was calculated using Equation (2).

(2) $$J=\frac{\Delta m}{\Delta t}*A$$

where *J* is water vapour flux, Δ*m* is the mass difference, Δ*t* is the time difference, and *A* is film area [84].

#### 3.2.8. Propolis Release Assay

A propolis release test on PCL + Prop samples was also carried out in saline solution (0.9% wt. NaCl), at 37 °C, 100 rpm, for up to 2 days (N = 3). Samples of 1 cm × 1 cm were placed in 2 mL of saline solution and collected at the following times: 0.5, 2, 24, and 48 h. The amount of propolis released was quantified on a UV-Vis Perkin Elmer Lambda 25 spectrophotometer (Boston, MA, USA) at 302 nm. The calibration curve is available in the Supplementary Materials (Figure S5).

#### 3.2.9. Scratch Wound Assay

Human gingival fibroblast (FGH) cell lines were obtained from Cell Bank of Rio de Janeiro, Brazil (Code 0089, information about this cell line: https://bcrj.org.br/celula/0089, accessed on 5 July 2022). Cell lines (2 × 10^5^ cells) were seeded in 24-well plates and allowed to attach for 24 h at 37 °C. Simulated wounds were created in confluent cells using a pipette tip. Cells were then rinsed with a medium to remove floating cells and debris. After washing, cells were cultivated in the presence of electrospun PCL + Prop mats for another 24 h. The culture plates were incubated at 37 °C. Cells cultivated without any film were used as the control. Wound gaps were measured at 0 and 24 h using an inverted microscope equipped with a digital camera. The percentage of wound healing was determined as the difference between the gap lengths according to Equation (3) [85].

(3) $$\begin{array}{l} \%\;Wound\;closure\\ =Average\;of\left(\frac{{\left[Gap\;length\right]}_{0h}-{\left[Gap\;length\right]}_{24h}}{{\left[Gap\;length\right]}_{0h}}\right) \end{array}$$

## 4. Conclusions

This work investigated the preparation, characterisation, and in vitro behaviour of electrospun films formed by PCL fibres, beaded fibres, and beads containing alcoholic propolis extract, aiming to be applied as wound dressings. An eco-friendly solvent system composed of acetic acid/formic acid was employed as a spinning solvent. Phytochemical analyses and FTIR confirmed the presence of active compounds in propolis; the extract was effectively encapsulated in the PCL fibres and acted as a lubricating agent, as seen in the viscosity analyses. The fibrillar nanostructured mats showed diverse morphology variations attributed to the hydrolysis of the ester bonds of PCL induced by the solvent that decreased the polymer molecular weight by approximately half every seven days of storage time. The PCL hydrolysis also influenced the degree of crystallinity and crystal structure on the samples, allowing an increase in the crystallinity, regardless of the presence of propolis, especially in films with beads. All mats presented a water vapour transpiration rate in an ideal range, compatible with values of commercial wound dressing products. The incorporation of propolis alcoholic extract significantly increased the mats’ hydrophilicity compared to PCL structures without propolis, which could promote a better environment to support cellular events during the wound-healing process. Furthermore, although the presence of beads in these nanostructures did not alter the burst release pattern, structures with beaded fibres displayed better results in the wound-healing assay with human gingival fibroblast (FGH) cells, accelerating the healing in 24 h. Due to the high surface area attributed to the fibrillar nanostructure and the presence of propolis extract, these films show good potential in improved wound dressing applications, encouraging further in vitro biological experiments to evaluate the growth and activity of keratinocytes and fibroblasts and even in vivo experiments.

## Figures and Tables

**Table 1:** Optimal values for sample production.

Variable	Fibres	Beaded Fibres	Beads
PCL Concentration, % (*w*/*v*)	30	30	30
Distance needle tip/collector, cm	10	10	10
Flow Rate, mL/h	2.0	1.0	1.0
Voltage, kV	10	15	15
Storage at 35 °C, days	0	7	14

**Table 2:** Fibre and bead diameter measured in SizeMeter 1.1 software.

Solution Sample	Storage Time (Days)	Morphology	Diameter	
			Fibre (nm)	Bead (μm)
PCL	0	Fibres	280.5 ± 70.1	
	7	Beaded Fibres	190.7 ± 83.7	5.6 ± 2.4
	14	Beads		2.9 ± 0.5
PCL + Prop	0	Fibres	208.3 ± 72.6	
	7	Beaded Fibres	159.2 ± 52.6	2.6 ± 0.7
	14	Beads		1.9 ± 0.5

**Table 3:** Thermal transitions refer to the heating and cooling cycles of PCL and PCL + Prop for the different morphologies.

Samples		First Heat Cycle			Second Heat Cycle		
		T_m_ (°C)	ΔH_m_ (J/mg)	X_c_ (%)	T_m_ (°C)	ΔH_m_ (J/mg)	X_c_ (%)
PCL	Fibres	68.4	26.4	17.4	57.0	27.1	17.9
	Beaded Fibres	71.1	51.2	33.8	62.0	38.3	25.2
	Beads	65.7	65.8	43.4	61.4	54.9	36.2
PCL + Prop	Fibres	71.4	31.3	20.6	64.3	28.0	18.5
	Beaded Fibres	67.3	54.4	35.9	62.0	42.0	28.0
	Beads	64.5	65.5	43.2	58.0	54.0	36.0

**Table 4:** Water vapour transpiration rate calculated for every sample with morphological variations from the water vapour flow (*J*).

Samples		*J* = Δ*m*/Δ*t* (mg/min)	Thickness (mm)	Water Vapour Transpiration Rate (g/m^2^ per day)
PCL	Fibres	0.0107	0.09	2179.84
	Beaded Fibres	0.0081	0.56	1650.16
	Beads	0.0088	0.48	1792.77
PCL + Prop	Fibres	0.0082	0.24	1670.53
	Beaded Fibres	0.0087	0.39	1772.39
	Beads	0.0086	0.14	1263.08

**Table 5:** Diameters before and after swelling and propolis release. Fibre and bead diameter after release assay measured in SizeMeter 1.1 software.

Samples	Diameters			
	Fibre (nm)		Bead (um)	
	Before	After	Before	After
PCL Fibres	280.5 ± 70.1	811.1 ± 381.0		
PCL Beaded Fibres	190.7 ± 83.7	461.2 ± 220.9	5.6 ± 2.4	6.6 ± 2.6
PCL Beads			2.9 ± 0.5	5.5 ± 1.6
PCL + Prop Fibres	208.3 ± 72.6	1098.5 ± 478.3		
PCL + Prop Beaded Fibres	159.2 ± 52.6	349.8 ± 109.9	2.6 ± 0.7	2.9 ± 0.9
PCL + Prop Beads			1.9 ± 0.5	

**Table 6:** Main electrospinning variables evaluated.

Variable	Value	
PCL Concentration	30	% (*w*/*v*)
Distance needle tip/collector	10	cm
Flow Rate	1.0–2.0	mL/h
Voltage	10–15	kV
Molecular Weight	26,500–100,517	g/mol
Humidity	50–70	%

## Data Availability

Not applicable.

## Supplementary Materials

The following supporting information can be downloaded at: https://www.mdpi.com/article/10.3390/molecules27165351/s1, Figure S1: Phytochemical qualitative analyses for flavonoids and phenolic compounds in propolis alcoholic extract; Figure S2. Fibre morphology obtained from 0-day storage solution electrospinning. Respectively: (A,C) Higher magnification scanning electron microscopy image for PCL and PCL + Prop electrospun mats; (B,D) Histograms of fibre diameters for PCL and PCL + Prop electrospun mats; Figure S3. Beaded fibre morphology obtained from 7-day storage solution electrospinning. Respectively: (A,D) Higher magnification scanning electron microscopy image for PCL and PCL + Prop electrospun mats; (B,E) Histograms of fibre diameters for PCL and PCL + Prop electrospun mats; (C,F) Histograms of bead diameters for PCL and PCL + Prop electrospun mats; Figure S4. Bead morphology obtained from 14-day storage solution electrospinning. Respectively: (A,C) Higher magnification scanning electron microscopy image for PCL and PCL + Prop electrospun mats; (B,D) Histograms of bead diameters for PCL and PCL + Prop electrospun mats; Figure S5. Propolis alcoholic extract calibration curve for propolis release assay. Absorbance at 302 nm; Figure S6. Histogram of morphologies by electrospun mats after swelling assay (Figure 9 in manuscript). Fibre Mats: (A) PCL, (B) PCL + Prop. Beaded Fibre mats: (C) Fibres PCL; (D) Fibres PCL + Prop, (E) Beads PCL, (F) Beads PCL + Prop, and Bead Mats (G) PCL.
