# Supplementary material for: Nanostructured Electrospun Polycaprolactone—Propolis Mats Composed of Different Morphologies for Potential Use in Wound Healing
Source: Molecules. 2022 Aug 22;27(16):5351. doi: 10.3390/molecules27165351 (PMC9413572; doi:10.3390/molecules27165351)
Supplement: Supplementary file 1 [file molecules-27-05351-s001.zip › molecules-1821137-supplementary.pdf]

Supplementary Materials

Nanostructured Electrospun Polycaprolactone—Propolis Mats Composed of Different Morphologies for Potential Use in Wound Healing

Agnes Chacor de Figueiredo [1], Javier Mauricio Anaya Mancipe [1,2], Aline Oliveira da Silva de Barros [3,4], Ralph Santos-Oliveira [3,4], Marcos Lopes Dias [2] and Rossana Mara da Silva Moreira Thiré [1,*]

[1] COPPE/Program of Metallurgical and Materials Engineering—PEMM, Universidade Federal do Rio de Janeiro, Rio de Janeiro 21941-599, RJ, Brazil

[2] Institute of Macromolecules Professora Eloisa Mano—IMA, Universidade Federal do Rio de Janeiro, Rio de Janeiro 21941-598, RJ, Brazil

[3] Brazilian Nuclear Energy Commission, Nuclear Engineering Institute—IEN, Rio de Janeiro 21941-906, RJ, Brazil

[4] Laboratory of Radiopharmacy and Nanoradiopharmaceuticals, Universidade Estadual da Zona Oeste, Rio de Janeiro 23070-200, RJ, Brazil

* Correspondence: rossana@metalmat.ufrj.br; Tel.: +55-21-3938-8500

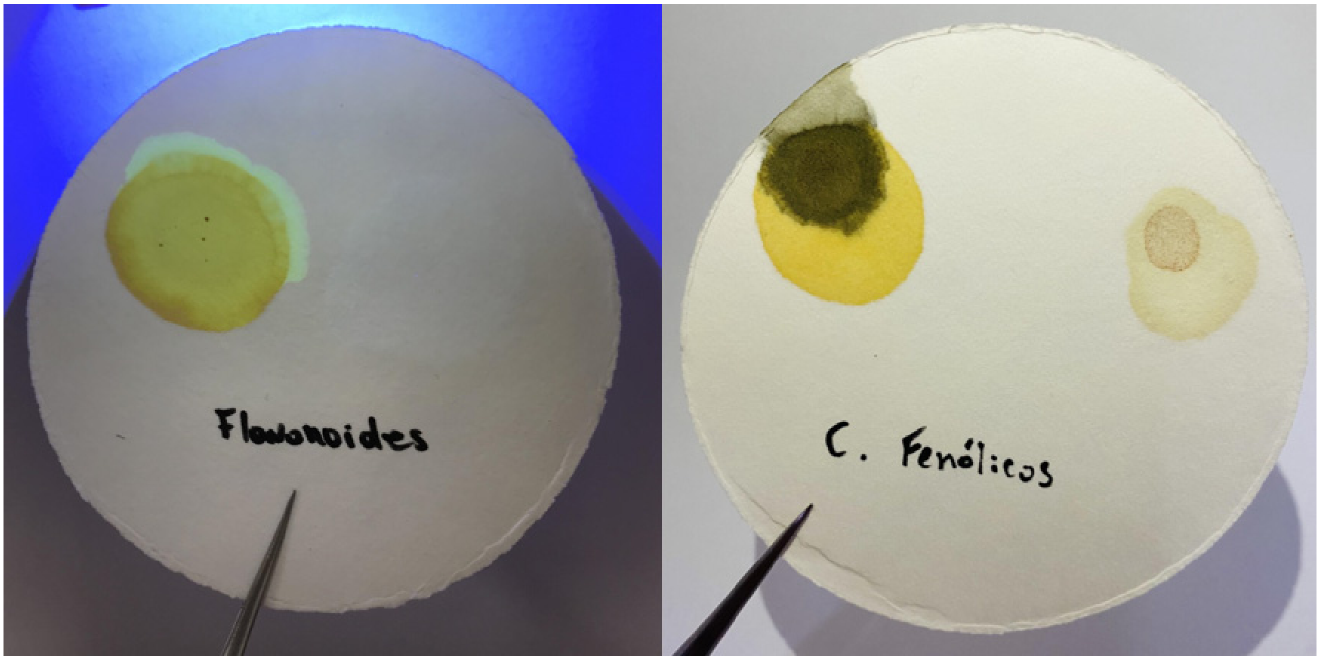

Figure S1. Phytochemical qualitative analyses for flavonoids and phenolic compounds in propolis alcoholic extract.

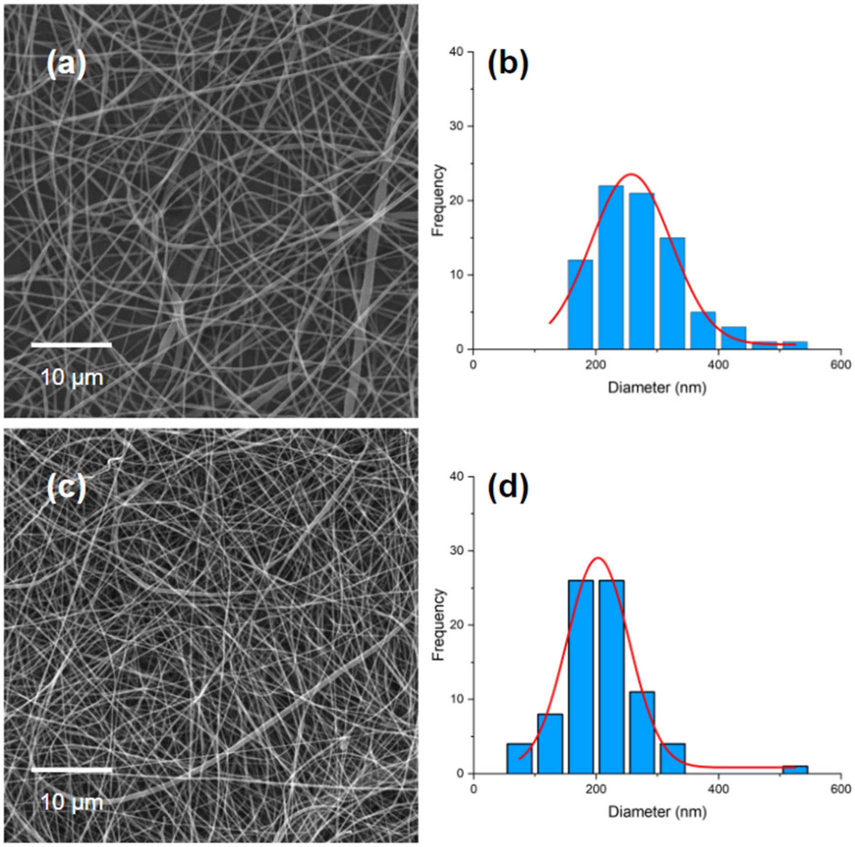

Figure S2. Fibre morphology obtained from 0-day storage solution electrospinning. Respectively: (**A**,**C**) Higher magnification scanning electron microscopy image for PCL and PCL + Prop electrospun mats; (**B**,**D**) Histograms of fibre diameters for PCL and PCL + Prop electrospun mats.

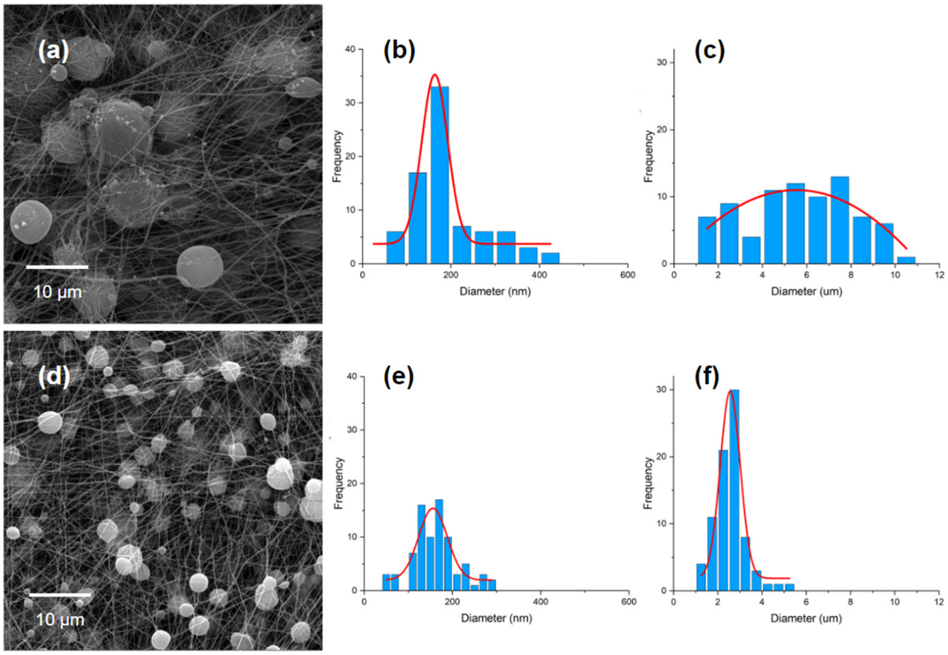

Figure S3. Beaded fibre morphology obtained from 7-day storage solution electrospinning. Respectively: (**A**,**D**) Higher magnification scanning electron microscopy image for PCL and PCL + Prop electrospun mats; (**B**,**E**) Histograms of fibre diameters for PCL and PCL + Prop electrospun mats; (**C**,**F**) Histograms of bead diameters for PCL and PCL + Prop electrospun mats.

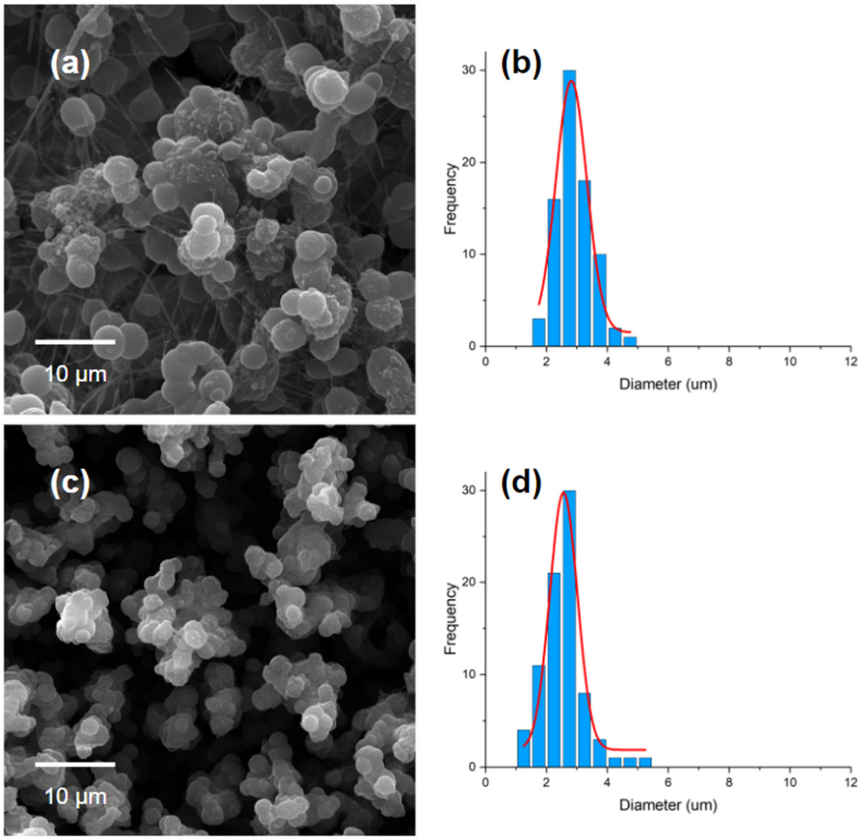

Figure S4. Bead morphology obtained from 14-day storage solution electrospinning. Respectively: (**A,C**) Higher magnification scanning electron microscopy image for PCL and PCL + Prop electrospun mats; (**B,D**) Histograms of bead diameters for PCL and PCL + Prop electrospun mats.

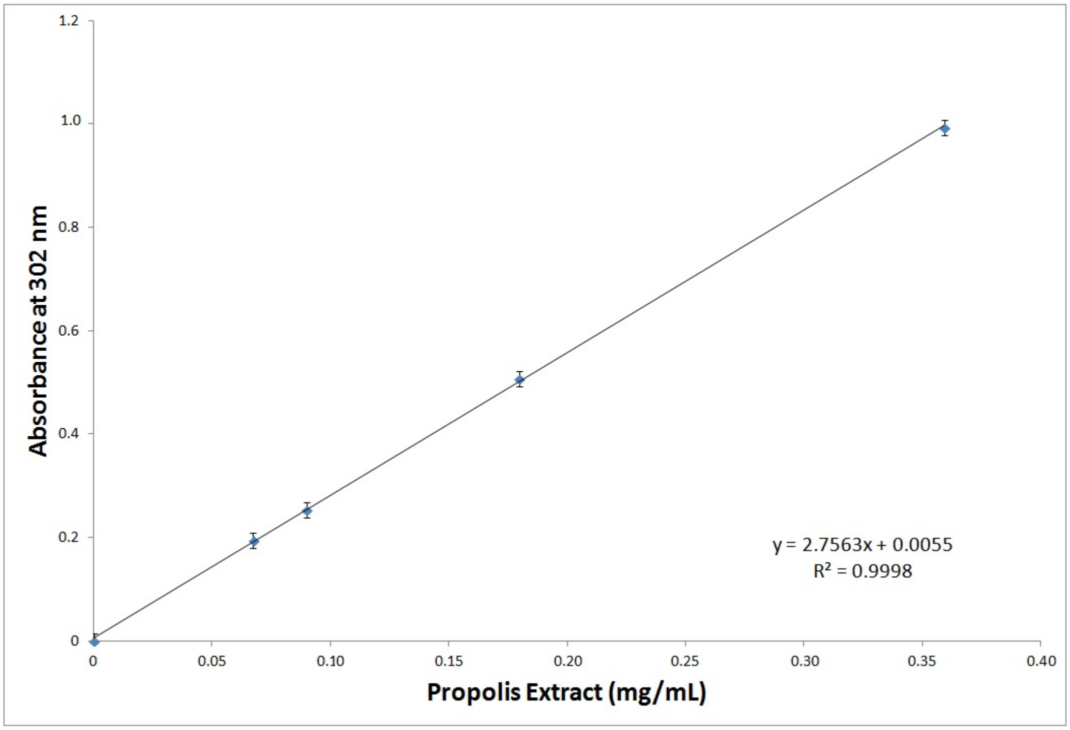

Figure S5. Propolis alcoholic extract calibration curve for propolis release assay. Absorbance at 302 nm.

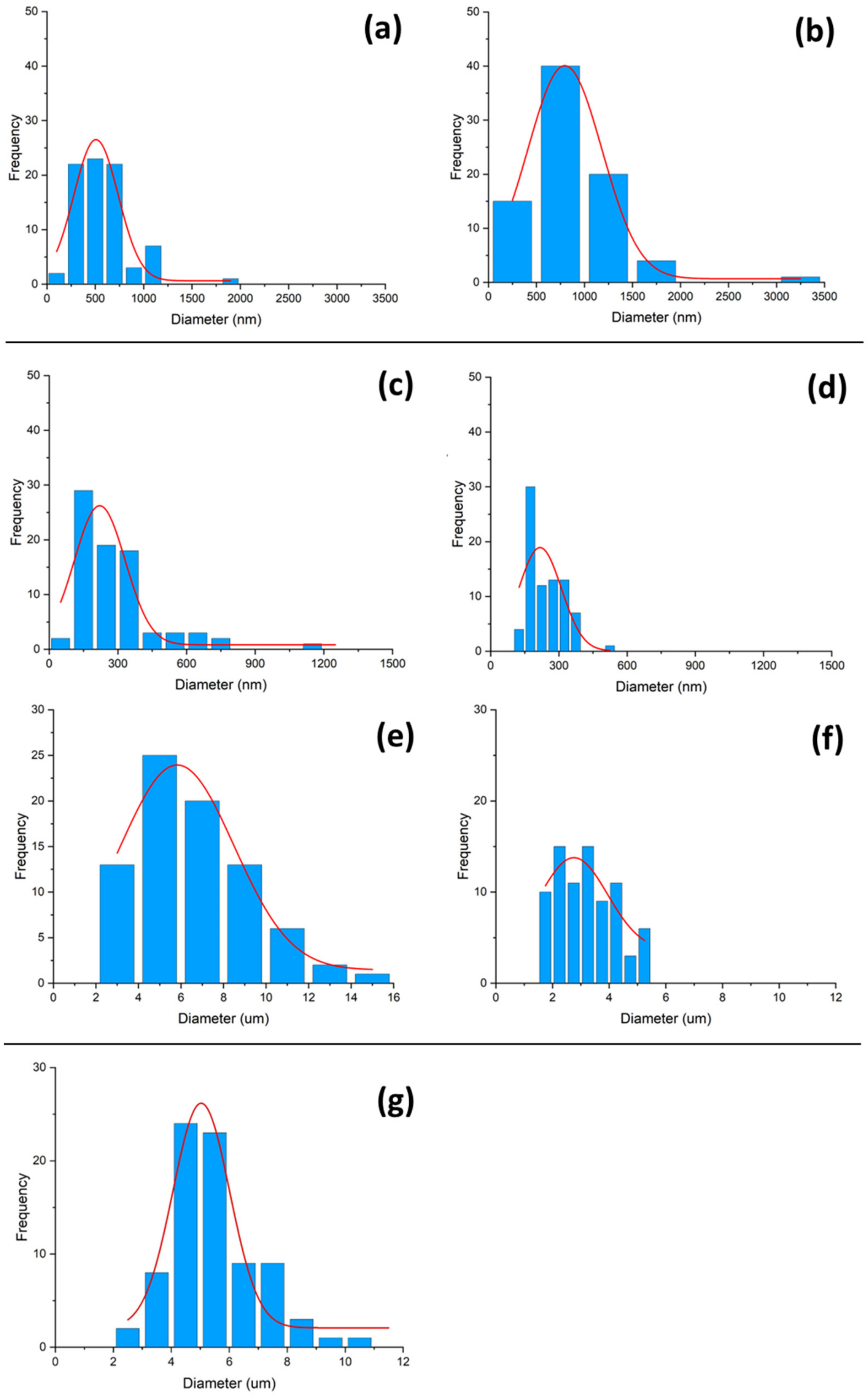

Figure S6. Histogram of morphologies by electrospun mats after swelling assay (Figure 9 in manuscript). Fibre Mats: (**A**) PCL, (**B**) PCL + Prop. Beaded Fibre mats: **(C)** Fibres PCL; (**D)** Fibres PCL + Prop, (**E**) Beads PCL, (**F**) Beads PCL + Prop, and Bead Mats (**G**) PCL.
